# An Evaluation Framework for Defining the Contributions of Telestration in Surgical Telementoring

**DOI:** 10.2196/ijmr.2611

**Published:** 2013-07-25

**Authors:** Andrius Budrionis, Knut Magne Augestad, Hiten RH Patel, Johan Gustav Bellika

**Affiliations:** ^1^Faculty of Science and TechnologyDepartment of Computer ScienceUniversity of TromsøTromsøNorway; ^2^University Hospital of Northern NorwayTromsøNorway; ^3^Virtual Surgical Skills Simulation CentreInstitute of CancerQueen Mary University of LondonLondonUnited Kingdom

**Keywords:** telementoring, telestration, annotation, impact, benefits, theoretical models, evaluation framework

## Abstract

**Background:**

An increasing quantity of research in the domain of telemedicine show a growing popularity and acceptance of care over distance systems among both clinicians and patients. We focus on telementoring solutions, developed for providing remote guidance to less experienced surgeons. Telestration is often regarded as an extra functionality of some telementoring systems. However, we advocate that telestration must be viewed as a core feature of telementoring due to its advantages.

**Objective:**

To analyze and define concepts, parameters, and measurement procedures to evaluate the impact of using telestration while telementoring.

**Methods:**

A systematic review of research dealing with telestration during remote guidance sessions was performed by querying three major online research databases (MEDLINE, Association of Computing Machinery, and Institute of Electrical and Electronics Engineers) using a predefined set of keywords (“laparoscopy”, “annotate”, “telestrate”, “telestration”, “annotation”, “minimally invasive”, and “MIS”).

**Results:**

The keyword-based search identified 117 papers. Following the guidelines for performing a systematic review, only 8 publications were considered relevant for the final study. Moreover, a gap in research defining the impacts of telestration during telementoring was identified. To fill this niche, a framework for analyzing, reporting, and measuring the impacts of telestration was proposed.

**Conclusions:**

The presented framework lays the basics for the structured analysis and reporting of telestration applied to telementoring systems. It is the first step toward building an evidence knowledge base documenting the advantages of live video content annotation and supporting the presented connections between the concepts.

## Introduction

The shift from open to laparoscopic surgery presents a fertile ground for an expansion of telemedicine (a set of medical practices without direct physician-patient interaction that are often realized via interactive audio-video communication [1]). Advances in information and communication technologies have resulted in the development of relatively low cost and high reliability devices as a medium for telemedical solutions. The demand for telementoring systems in laparoscopic surgery, comprising the real-time interactive teaching of techniques by an expert surgeon to a student at a distant site [1], in laparoscopic surgery was partially motivated by the decreasing ratio of general practice surgeons to population, a problem that is predicted to increase in the future [2]. Studies have shown the benefits of using telementoring, especially in the field of laparoscopic surgery [3,4]. Time and cost reductions as well as a more efficient surgical education are only a few of the many, mostly evident advantages of this developing technology [3].

Telementoring has been discussed extensively in the literature, with multiple solutions demonstrated for laparoscopic surgery [5,6]. Its place in surgery has been described by Doarn [7] as “a natural fit”. Furthermore, videoconferencing has gained increasing popularity in all fields of medicine, especially in follow-up and out-patient treatment [8-10].

In spite of the recent advances in mobile devices, the current body of literature tends to limit the telementoring approach to stationary platforms. However, the Mobile Medical Mentor project team advocates using mobile devices for telementoring due to additional benefits compared to the stationary ones [11]. The fact that mentors rarely stay at one location and are highly mobile should be sufficient impetus for further exploration of mobile platforms for telementoring. Having a telementoring device within reach should increase the availability of experts. Therefore, the main challenge is to ensure that the required telementoring infrastructure is available on a mobile medium [11].

**Figure 1:**
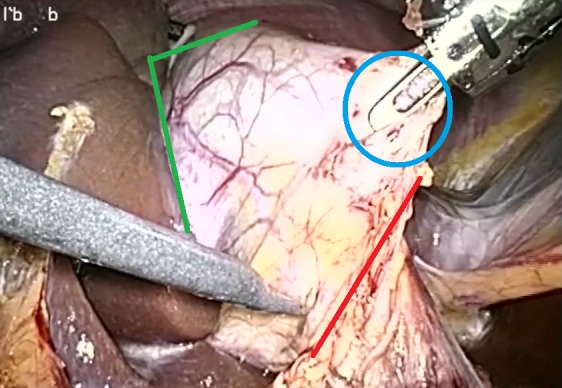
Surgical telestration.

Telestration is defined as a technique for enabling the drawing of freehand markups (annotations) over image or video (Figure 1). Although it is mostly used in commenting on sports and weather forecasts, it often attracts the attention of medical personnel [12,13]. In the domain of telemedicine, telestration is normally considered to be an additional accessory function of telementoring systems. As the overall impacts and benefits of telestration are not clear, this paper aimed to analyze the available studies reporting the use of telestration to form a systematic assessment of the reported outcomes of using telestration in minimally invasive surgery (MIS).

The paper is structured as follows: after a short introduction to the developing domain of telemedicine, the motivation for using telementoring systems is presented. The telestration feature is analyzed in greater detail.

The Methods Section defines the procedures that were followed to summarize the current body of knowledge in using telestration and guidelines to develop the evaluation framework. The Results Section represents the achievements of a systematic review procedure and highlights the identified gap in the available research. The following section discusses the identified niche in research, introducing an evaluation framework for analyzing, measuring, and reporting the impacts of using telestration during telementoring sessions. Evaluation biases and confounders were analyzed in the Discussion Section.

## Methods

### Overview

This section is divided into two sub-sections, the first of which describes the search process for relevant research. This search was performed to summarize the available body of knowledge in evaluating the impacts of telestration applied to telementoring systems. The second part studies the development of an evaluation framework in more detail by introducing the methodology for developing theoretical models.

### Systematic Literature Review

To form a solid basis for this research, a systematic literature review of telementoring applications that include a telestration feature in MIS was performed. The review was carried out following the guidelines defined by Kitchenham [14]. The aim of reviewing the available body of literature in the field was motivated by the need for a summarized and structured evidence knowledge base. Moreover, we intended to identify the gaps in current research and develop guidelines for further investigation of the domain [14,15].

The search for related studies was limited by publication date (1992-2012) due to technological progress. Search results (published before 1992) were considered too old to be relevant. Three major online research databases (MEDLINE, Association of Computing Machinery [ACM], and Institute of Electrical and Electronics Engineers [IEEE]) were selected as data sources for the review. The search was performed on the 13th of November, 2012. The selected databases were queried using the same set of keywords, containing combinations of the following terms: “laparoscopy”, “annotate”, “telestrate”, “telestration”, “annotation”, “minimally invasive”, and “MIS”. Keywords were selected based on earlier research in the domain [3,11,12], in accordance with the corporate decision of the co-authors. The results of the search were analyzed by one reviewer to determine the eligibility of the studies. We admit the possible weakness of including only one reviewer in the study selection process; however, due to the narrow focus and straightforward evaluation of the papers, this bias should be minimal.

### Developing the Evaluation Framework

The methodology presented by Sjøberg et al [16] was employed to develop the theoretical fundamentals for evaluating the impacts of the technology. The initial focus was to define the basic elements of the theory-constructs. We aimed to use concrete and easily measurable concepts instead of abstractions. This approach makes the framework less universal; however, our purpose was to develop a specialized evaluation methodology for a narrow field telestration system. Relationships between the concepts were defined to highlight the existing dependencies. The framework was supplemented by explanations and a description of the scope. Constructs, relationships, and explanations are the main components for developing theoretical models [16].

## Results

### Systematic Literature Review

**Figure 2:**
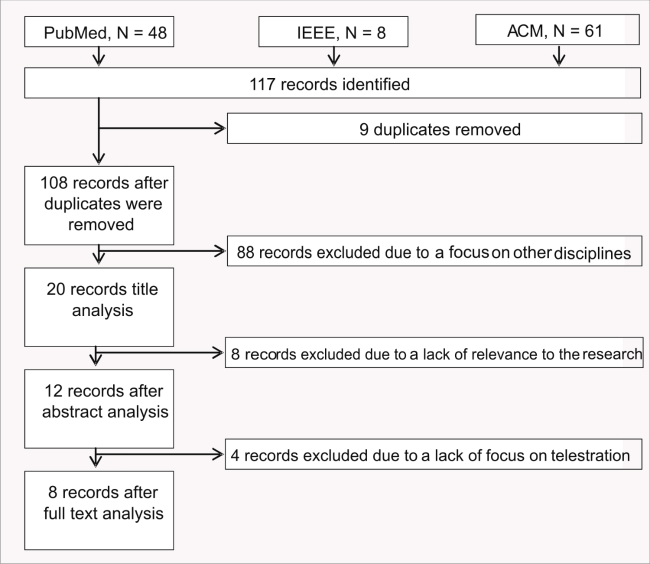
Systematic review flow diagram.

The keyword-based search resulted in 117 papers in total, as shown in Figure 2. We aimed to identify and analyze papers reporting the use of telestration feature. The main focus was on the analyses of the impacts of telestration to the overall procedure toward research-based proof of the benefits for telementoring. Nine instances were discarded as duplicates. Title analysis resulted in 88 exclusions due to a focus on other disciplines. An additional 8 records were discarded after analyzing the abstracts of the papers due to a lack of relevance to the current research topic. Only 12 (10%) studies were considered eligible for the final stage-full text analysis, which revealed that only 8 papers were relevant for the current study: [13,17-23] (4 papers were excluded due to lack of focus on telestration)*.*

The review was performed to assess the impacts and benefits of telestration as applied to telementoring systems and then analyze these data in a systematic manner. The study revealed a gap of knowledge in assessing the outcomes of telestration. All of the selected studies reported the use of telestration; however, no analysis or assessment of its impacts was identified. The only outcome was a significant improvement of the mentoring session due to the ability to annotate graphic content, reported in 3 papers [18,20,21]. No support for the claims was provided, making them sound subjective. The remainder of the papers declared the feature to be an integral part of the telementoring system; although, no additional details were made available.

The purpose of the review was to assess the potential benefits of telestration in telementoring based on reported experiences. However, we were unable to achieve our goal due to the lack of existing research. The shortage of publications motivated the need to propose an evaluation framework for analyzing the outcomes of telestration in the scope of telementoring.

### Definition of Concepts and Theoretical Models

An extra layer of interaction and complexity is added by providing the ability to annotate the graphic content used in a telementoring session. On the one hand, telestration simplifies pointing actions, eliminating the need for discussion to define the exact location in the visual representation; while on the other hand, it introduces extra complexity into the workflow model of the procedure. This complexity includes controlling of the telementoring system and annotations as well as extra hardware in the operating room. In this Section, we aim to define the potential benefits of telestration and discuss the impact of telestration on the outcome of the overall procedure.

**Figure 3:**
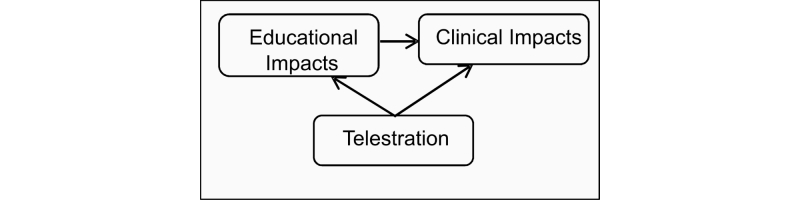
Impacts of telestration.

**Figure 4:**
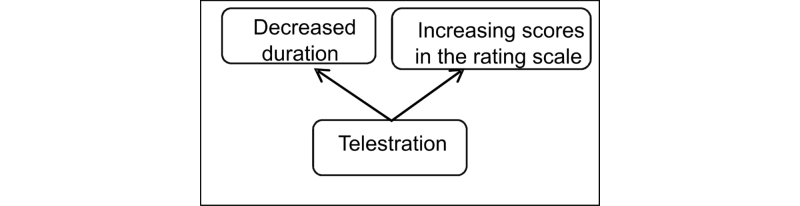
Educational outcomes of telestration.

To define the influence of telestration while telementoring, the outcomes were divided into Educational and Clinical Impact groups, highlighting the direct influence of the education process on potentially improved patient outcome (see Figure 3). To lay the basis for determining the impact of this feature, we focus on the case of laparoscopic surgery as an initial point for a video conferencing-based telementoring and surgical education. The laparoscopic procedure was selected because of its high visual component and the fact that the procedure is already transmitted to a monitor, which allows the easy adoption of the mentoring approach. As the local surgeon, hereafter referred to as mentee, is observing the progress of the procedure on the monitor, sharing this representation with a remote mentor is highly feasible. However, representation sharing and verbal interaction between endpoints (a common telementoring approach) is not sufficient in some cases. From an educational perspective (Figure 4) telestration is a feature that adds interactivity to the learning process, which should result in increasing quality of education through more comprehensive instructional material. An increased quality of education (defined quantitatively as increasing scores in the rating scale) should be enough impetus to suggest the possibility of a shorter duration of surgical training. This change could potentially lead to a rising number of highly experienced medical personnel and lower education costs. However, a lack of research leaves the assumptions poorly supported until the validation of the framework on real-life cases.

**Figure 5:**
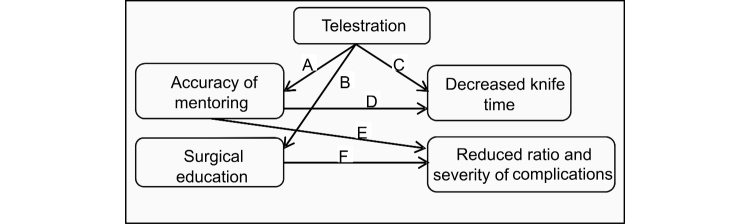
Clinical outcomes of telestration.

From a clinical perspective, the overall goal is the improved patient outcome, defined quantitatively as the ratio and severity level of complications, as shown in Figure 5. We assume that the use of the telestration feature in mentoring should result in increased accuracy of the surgical decisions, relation A in Figure 5, and overall mentoring process as the exact location of interest can be defined visually instead of more ambiguous verbal description. Secondly, the duration required to indicate the exact location in the operative field should decrease, resulting in a decreased duration of the overall procedure, relation C in Figure 5. We assume that following more accurate directions of the mentor should also decrease the duration of the procedure, relation D in Figure 5, help avoid clinical errors and shorten the recovery period, contributing to the overall goal–improved patient outcome, relation E in Figure 5. Moreover, shorter recovery and hospitalization is directly related to lower costs of treatment. The concept of “surgical education” represents the connection of educational and clinical outcomes, depicted in Figure 3, while relation B in Figure 5 summarizes the impacts of telestration with respect to improving surgical education as discussed above. F is one of the fundamental relations in the model dealing with the direct impact of improved education on the final well-being of the patient.

Another important aspect that should not be omitted is time consumption. From the perspective of the mentor, time savings contribute to a reduction of the workload as well as increased availability of the expert. The mentee should also experience decreased time consumption, resulting in shorter duration of the overall procedure. These issues pose the following question: given that the resources available in the hospital are constant, is it possible to treat more patients during the same period of time by decreasing the duration of the procedure? Moreover, the costs of the treatment should also diminish.

To summarize the Section, we assume that telestration is a feature that improves telementoring techniques. However, due to lack of studies on the possible impacts on the overall mentoring process, our claims remain poorly supported. Only the most obvious relations were discussed due to the simplicity of the models. Other dependencies may exist.

### Impact Measurement

To prove the hypotheses formulated in Section Definition of Concepts and Theoretical Models, a measurement system needs to be established. The purpose of such system is to assess if the introduction of a particular feature resulted in expected outcomes. This system calls for a comparison of measurements before and after the procedure workflow was supplemented by the new technology.

Evaluating the educational outcomes, shown in Figure 4, is straightforward, as the “decreased duration” and “increasing scores in the rating scale” concepts are easy to measure. The same strategy applies to relations C and D (“decreased knife time”) as well as E and F (“reduced ratio and severity of complications”), which refer to the clinical outcomes of telestration in Figure 5. The established Clavien-Dindo classification of surgical complications is proposed as a metric for assessing the grade of postoperative complications [24]. Relation B in Figure 5 represents the link between telestration and improved educational outcomes, depicted in Figure 3. The main challenge is to prove the dependency between using telestration and improved accuracy of mentoring (relation A, Figure 5). Although this dependency may appear self-evident, due to the increased accuracy of pointing actions, a reliable measure is complicated. Therefore, we propose a combined measure for determining the existence and benefits of this relationship. This measure assesses the following: (1) the number of guidance misinterpretations (possibly leading to errors), (2) the number of requests to clarify the advice, (3) the total time spent on guidance during the procedure, and (4) the number of mentoring interruptions to the flow of the procedure. The combined score is used to determine the accuracy of the mentoring accompanied by telestration. The measures are summarized in Table 1.

To evaluate our hypotheses, a comparison of measures before and after introducing the technology to the operating room must be performed. ALFA Toolkit [25] is one of the tools for analyzing the captured videos, including assessing the durations and other parameters. Results from similar research prove the feasibility of using the tool for the analysis of multi-channel videos in order to measure the impacts of the technology on the workflow of the procedure [26-28]. Three video sources (the output from the laparoscopic camera, the telementoring video and an overview of the operating room, which captures the actions of the surgical team as well as the laparoscopic and telementoring monitors) accompanied by an audio record will be used to evaluate the changes in the procedure workflow. The analysis requires a manual video coding to determine the start and end points of a particular event. This step may become ambiguous; therefore, it should be performed by multiple independent coders. Average value should be used in a case of coders’ disagreement, kappa coefficient should be reported. The following parameters should be encoded and measured using combined videos of the procedure:

- Start and end point of the procedure (knife time)
- Number of guidance misinterpretations
- Number of requests to clarify the guidance
- Start and end point of every telementoring interaction between mentor and mentee
- Initiator of each interaction

Duration and timing-based measures are automatically collected by ALFA Toolkit during the coding process, while the numerical data (number of misinterpretations and clarification requests, initiator of the interaction) is recorded by the coders in an MS Excel sheet, which is combined with the values exported from ALFA toolkit for further analysis. Average values together with dispersion coefficients are used to represent the final results.

**Table 1:** Measurement system.

Parameter	Relationship in Figure 5	What is measured?	Measure unit
Knife time	C	Duration: starting point–first incision, end point–end of the procedure.	Minutes, seconds
Ratio and severity level of complications	E, F	Number of complications / overall number of performed procedures and severity of the complications, if any.	Ratio coefficient and a grade in the Clavien-Dindo classification scheme for surgical complications [24]
Score in rating scale (surgical education)	B	Scores in the predefined scale to measure the progress of education.	Rating score
Duration (surgical education)	B	Duration required to reach the same level in the rating scale.	Hours
Accuracy of mentoring	A	1) Number of guidance misinterpretations (possibly leading to errors), 2) Number of requests to clarify the guidance, 3) Total time spent on guidance during the procedure, and 4) Number of interruptions of the flow of the procedure for mentoring.	Combined score

## Discussion

### Principal Findings

Research biases were analyzed in detail by Hartman et al [29] to increase the awareness of methods of mitigating their undesirable impacts. Although a number of biases were studied, in this phase of the project, the most attention is given to measurement-related biases.

An accurate measure of the parameters defined in Table 1 is a challenge. However, to achieve accurate results in each experiment, the requirements for the analyzed procedure and stakeholders should be formalized to keep them as standard as possible. Unfortunately, the observed procedures are never the same, even if a strictly predefined workflow is followed. Moreover, the human factor shapes the workflow according to the preferences and previous experiences of the surgeon in charge. Complete control of the mentioned factors may be impossible; however, to increase the accuracy, only very similar procedures are to be studied and represented in the final results. The similarity of the cases is based on preoperative data (medical imaging, observations, etc.). Moreover, it is assumed that the remote mentoring approach is also highly influenced by the social connection between mentor and mentee, their previous experiences in telementoring, and their current attitudes. An accurate determination of the levels of experience of mentor and mentee is also a subjective parameter influencing the final results of the study. Randomization of the observed procedures and surgical team may be a way to achieve more consistent results in this case.

From the point of view of the measurement process itself, ambiguity is also inevitable. To avoid biases in duration and score estimation based on video representation, only the consensus decision of the project team should be considered correct.

The presented framework addresses the theoretical side of the project. It was developed as a methodology section for the on-going research. Logical relations between the concepts are represented in order to understand the influence of the technology on procedural workflow and define it in a measurable manner. The research still suffers from the lack of evidence and validation on real life test cases.

To conclude the section, we admit the potential weaknesses of the proposed research caused by the mentioned biases. The list of the biases influencing the final outcome of the experiment is not exhaustive; however, we aimed to mention only the most obvious cases.

### Conclusions

The paper aimed to summarize the experience of using telestration during telementoring sessions to highlight the impacts of this feature on the mentoring process and workflow of the procedure. However, an absence of research reflecting the use of telestration was identified. Therefore, a framework for analyzing the impact of live video content annotation was proposed. To keep the models simple and adaptable, easy-to-measure concepts were employed and only the most obvious dependencies were discussed. To support our claims and the presented models, an impact measurement procedure was defined.

The presented framework and impact measurement procedures form a methodology for the further analysis and reporting of research on telestration and telementoring systems. Having a more formalized method should increase the quality and quantity of publications in this field, providing an evidence-based knowledge base supporting the development of the telementoring domain and the introduction of new technologies and features with the aim of improving patient outcomes.

## Abbreviations

ACM: Association of Computing Machinery
IEEE: Institute of Electrical and Electronics Engineers
MIS: minimally invasive surgery
